# Identifying the Role of E2 Domains on Alphavirus Neutralization and Protective Immune Responses

**DOI:** 10.1371/journal.pntd.0004163

**Published:** 2015-10-16

**Authors:** James Weger-Lucarelli, Matthew T. Aliota, Attapon Kamlangdee, Jorge E. Osorio

**Affiliations:** Department of Pathobiological Sciences, University of Wisconsin-Madison, Madison, Wisconsin, United States of America; University of Texas Medical Branch, UNITED STATES

## Abstract

**Background:**

Chikungunya virus (CHIKV) and other alphaviruses are the etiologic agents of numerous diseases in both humans and animals. Despite this, the viral mediators of protective immunity against alphaviruses are poorly understood, highlighted by the lack of a licensed human vaccine for any member of this virus genus. The alphavirus E2, the receptor-binding envelope protein, is considered to be the predominant target of the protective host immune response. Although envelope protein domains have been studied for vaccine and neutralization in flaviviruses, their role in alphaviruses is less characterized. Here, we describe the role of the alphavirus E2 domains in neutralization and protection through the use of chimeric viruses.

**Methodology/Principal Findings:**

Four chimeric viruses were constructed in which individual E2 domains of CHIKV were replaced with the corresponding domain from Semliki Forest virus (SFV) (ΔDomA/ΔDomB/ΔDomC/ ΔDomA+B). Vaccination studies in mice (both live and inactivated virus) revealed that domain B was the primary determinant of neutralization. Neutralization studies with CHIKV immune serum from humans were consistent with mouse studies, as ΔDomB was poorly neutralized.

**Conclusions/Significance:**

Using chimeric viruses, it was determined that the alphavirus E2 domain B was the critical target of neutralizing antibodies in both mice and humans. Therefore, chimeric viruses may have more relevance for vaccine discovery than peptide-based approaches, which only detect linear epitopes. This study provides new insight into the role of alphavirus E2 domains on neutralization determinants and may be useful for the design of novel therapeutic technologies.

## Author Summary

Chikungunya virus (CHIKV) is the cause of an ongoing explosive outbreak of arthritic disease in the Americas. Related alphaviruses cause human/animal disease globally, yet no vaccines or antivirals exist for human use. Although numerous candidate vaccines and therapies are being developed, little is known about the specific viral targets of an effective host immune response. Previous studies have used peptide or monoclonal antibody approaches, which can have serious limitations. Chimeric viruses between closely related species are proven tools to study a variety of viral characteristics. Using this approach, we developed a panel of viruses, which highlight the importance of the alphavirus domain B in protection in mice and serum neutralization in humans. Previous work on flaviviruses has shown that subunit approaches can be effective for vaccination and diagnostic purposes. Thus, the use of E2 domains as vaccine antigens and in clinical diagnostics for alphaviruses warrants further study.

## Introduction

Alphaviruses are a diverse group of arthropod-borne viruses (arbovirus) that are distributed worldwide [1]. Chikungunya virus (CHIKV) has been the cause of several recent outbreaks of arthritic disease and has now spread into the Caribbean and Central/South America, with at least 44 countries in the Americas having reported locally acquired cases, including the United States [2]. The disease caused by CHIKV is characterized by high fever and painful arthralgia, which can last for months or even years after infection [3]. The primary mosquito vector for CHIKV transmission is *Aedes aegypti;* however, recent evolution of certain lineages of the virus has allowed increased transmission by the more temperate *Aedes albopictus* [4, 5]. While this adaptation has facilitated recent outbreaks of CHIKV in Europe and southeast Asia, the virus circulating in the Americas does not possess this mutation [6]. Still, recent work studying CHIKV evolution has shown that emergence of adaptive mutations, which increase transmissibility in *Ae*. *albopictus* can occur in just one passage [7] putting more temperate countries, like the United States, at considerable risk. Other alphaviruses such as the equine encephalitis viruses (eastern, western and Venezuelan), O’nyong nyong (ONNV), Sindbis (SINV) and Semliki Forest (SFV) viruses, also pose a considerable threat to human and animal health around the globe [8].

CHIKV, like all alphaviruses, has a positive sense single stranded RNA genome. The non-structural proteins (nsPs; nsp1-nsp4) constitute the 5’ end of the genome and the 3’ end consists of structural proteins (sPs; C, E3/E2, 6K/E1) produced through a sub-genomic RNA (Reviewed in [9]). The two envelope proteins, E1 and E2, interact closely on the surface of the infectious virion and perform membrane fusion and receptor binding functions, respectively [10]. The alphavirus E2 protein consists of three distinct domains (A, B, and C), and E2 has been previously implicated as the major target of the host immune response [11–14]. But little is known about the individual role of any of the three distinct domains in alphavirus immunity. In contrast, flavivirus envelope protein domains have been extensively studied and are being exploited for use in understanding the host immune response and as vaccine antigens [15, 16]. While a considerable amount of knowledge has led to a greater understanding of the host immune response to a variety of alphaviruses, this has not resulted in any licensed human vaccines. Although many promising candidate vaccines exist for CHIKV [17–20], safety concerns, particularly with live virus vaccines, are considerable. Consequently, we recently showed that a poxvirus vectored vaccine expressing CHIKV E2 provided 100% protection in highly immunocompromised mice [21], suggesting safer subunit vaccines could be viable alternatives. Still, little is known about the specific viral targets of an effective host immune response.

Accordingly, we assessed the role of the alphavirus E2 domains in protection and immunogenicity using chimeric viruses. Chimeric viruses are useful to assess the function of proteins or protein domains in related viruses and have been instrumental in unraveling determinants in host range, tissue tropism, and virulence [22–25]. We constructed four chimeras, each of which had E2 domains from CHIKV replaced with the corresponding region from SFV (ΔDomA/ΔDomB/ΔDomC/ΔDomA+B). CHIKV and SFV, both members of the SFV complex of Old World alphaviruses, are sufficiently similar to produce viable chimeras (Weger-Lucarelli *et al*. in revision). Despite their similarity, it has previously been shown that SFV was not neutralized by anti-CHIKV serum [26]. Herein, through live-virus and UV inactivated vaccination approaches, we showed that domain B was the primary determinant of neutralization for these viruses and also was critical in the development of neutralizing antibodies, in mice. Neutralization studies with sera from human patients previously infected with CHIKV confirmed this trend, as CHIKV containing SFV domain B showed reduced neutralization capacity.

## Methods

### Ethics statement

This study was carried out in strict accordance with the recommendations in the Guide for the Care and Use of Laboratory Animals of the National Institutes of Health. The IACUC protocol (Protocol #V01380) was approved by the Institutional Animal Care and Use Committees of the University of Wisconsin-Madison. Manipulation of cDNA clones and virus constructs was approved under IBC SC# 12-077R at the University of Wisconsin-Madison. Human samples (a kind gift from Dr. Juan Carlos Dib) were collected in Santa Marta, Colombia under approval of the ethics committee of the Fundación Salud Para el Trópico (#042014).

### Cell culture

Baby hamster kidney cells (BHK-21; ATCC # CCL-10) were maintained in high glucose Dulbecco's modified Eagle medium (DMEM) supplemented with 10% fetal bovine serum, nonessential amino acids, sodium pyruvate, 10mM HEPES and penicillin-streptomycin and incubated at 37°C in 5% CO2.

### Construction of cDNA clones

CHIKV strain SL-CK1, courtesy of Dr. Scott Weaver (University of Texas Medical Branch), and SFV strain L10, a gift from Dr. John Fazakerley (The University of Edinburgh), were used for all experiments. Infectious cDNA clones of each virus were constructed using overlap extension PCR [27] into a plasmid backbone which employed a CMV promoter for production of viral genomic RNA, courtesy of Dr. Brian Geiss (Colorado State University), circumventing the need for RNA production [28]. CHIKV/SFV chimeras were constructed in the same manner, except CHIKV was used as the backbone, replacing the native E2 domains with those from SFV (chimeras herein called ΔDomA/ΔDomB/ΔDomC/ΔDomA+B). Each virus contained the green fluorescent protein (GFP) expressed through the inclusion of a repeated sub-genomic promoter for visualization of infection, 5’ to the structural polyprotein. Primers and sequences are available upon request.

### Sequencing

For all viruses generated, the entire genome was sequenced from the cDNA clone. In addition, the entire structural poly-protein was sequenced for all virus stocks used in all experiments, maintained 100% match with cDNA sequence. Sequencing was performed by first extracting viral RNA using the ZR Viral RNA kit (Zymo Research, Irvine, CA). Reverse-transcription was then performed to produce cDNA using the Superscript III Reverse-Transcriptase First-Strand Synthesis System (Invitrogen, Carlsbad, CA). cDNA was then used as a template for PCR with virus-specific primers using Q5 high fidelity polymerase (New England Biolabs, Ipswich, MA). The amplicons were then used for sequencing at the Biotech Center located at the University of Wisconsin-Madison. Vector NTI (version 11.5, Invitrogen) was used to align and assemble sequencing data.

### Electroporation and virus production

Transfection grade plasmid was prepared using a column based maxi-prep kit from Zymo Research. For virus recovery, two μg of purified plasmid was electroporated into BHK-21 cells using a BioRad Gene Pulser (Hercules, CA). Briefly, 80–90% confluent T175 flasks of BHK-21 cells were trypsinized and washed twice in PBS, followed by one wash in cytomix buffer [29]. Cells were then resuspended in 500 μL cytomix buffer with ATP and glutathione, followed by addition of plasmid, this mixture was then transferred to a 2mm cuvette. Cells were electroporated using infinite resistance, 300V and 960 μF capacitance. Following electroporation, cells were plated in T175 flasks. Virus was harvested 24–72 hours post-electroporation (depending on the virus) and cellular debris was removed at 2000xg. Virus was concentrated by overnight centrifugation at 4°C at 13,500xg. The pelleted virus was then resuspended in TEN buffer and stored in small volume single-use aliquots at -80°C. Infectious virus was titered by plaque assay on BHK-21 cells.

### Virus inactivation

Prior to UV-inactivation, all chimeras were propagated in BHK-21 cells at a multiplicity of infection (MOI) of 0.1 PFU/cell. The supernatant was harvested 24 hours following infection and infectious titer was determined via plaque assay on BHK-21 cells. Supernatant was then subjected to UV-inactivation using two exposures to 5 x 10^5^ μJ in a Stratalinker 1800 UV Crossover (Stratagene, La Jolla, CA) based on previous reports [17]. Complete inactivation was confirmed via infection of BHK-21 cells; since each virus expressed GFP, viable virus could be readily observed via fluorescence microscopy. Following complete inactivation, viruses were concentrated using a 100kD cut-off filter (Millipore, Darmstadt, Germany) roughly 50-fold and protein concentration was normalized to 5 mg/mL using a BioRad protein assay with bovine serum albumin (BSA) standard. Additional confirmation of inactivation was performed at this point by infection of BHK-21 cells.

### Animal studies

For live-virus experiments, groups of six-week-old male C57bl/6 mice (Jackson Laboratories, Bar Harbor, Maine) were infected intradermally (ID) in the left hind footpad with 10^5^ PFU of each virus (CHIKV, SFV, ΔDomA, ΔDomB and ΔDomC). ΔDomA+B was not included in live virus experiments because it was highly attenuated in cell culture. All live virus experiments used virus direct from electroporation. Vaccination with inactivated virus was performed with 5 μg of total protein with no adjuvant via the same route. Mice receiving inactivated virus were boosted 28 days post-prime with the same dose. Mice were bled prior to challenge to assess humoral responses.

Two months post initial vaccination, mice were challenged with 10^5^ PFU of wild-type SFV or CHIKV ID in the left hind footpad (excluding the group that received live SFV, which succumbed to infection) and monitored for morbidity and mortality. Infected mice were bled via maxillary vein at different times post-infection and viremia was assessed by TCID_50_ [30]. Mice vaccinated with live virus were only challenged with SFV, as previous reports have shown that anti-SFV serum is protective against CHIKV, but not the converse [26]. Morbidity was assessed post-infection by weight loss and footpad swelling. Footpad measurements were taken with a digital caliper as the height of the hind feet at the balls. Subsets of mice were euthanized at different days post-infection and organs were harvested and fixed with 4% PBS buffered paraformaldehyde. Tissues for paraffin embedding were submitted to the Histology Laboratory at the School of Veterinary Medicine at the University of Wisconsin-Madison, where they were processed and sectioned before staining with hematoxylin and eosin (H&E).

### Antibody titers

Assessment of neutralizing antibodies in mice was performed using a modified luciferase based assay (described in [31]). CHIKV and SFV clones expressing NanoLuc luciferase (nLuc) (Promega, Madison, WI) in-frame between the capsid and E3 proteins were engineered as previously described [32]. Neutralization was performed by incubating heat-inactivated serum diluted 1:20 with 5x10^3^ PFU of either CHIKV or SFV expressing nLuc overnight at 4°C. Confluent BHK-21 in 96 well plates were then infected in triplicate with serum: virus mixture for one hour, followed by washing and addition of fresh media. Five hours post-infection, media was discarded and cells were lysed and analyzed for luciferase expression using the Nano-Glo luciferase system (Promega). Data are expressed as fold-neutralization using normal mouse serum for normalization. Plaque reduction neutralization 50% (PRNT_50_) assay in BHK-21 cells was used for determination of neutralizing titers in human samples (described in [33]).

### Statistics

Statistical analyses were run using GraphPad Prism software version 6 (San Diego, CA). Replication, viral load and neutralization data were analyzed using the student’s *t* test. Variances were compared using the F test. Survival analyses were performed using Kaplan-Meier curves with the log rank test. An alpha of 0.05 was used for all studies as the threshold for significance. All experiments were repeated at least twice with consistent results.

## Results

### Genome organization and construction of CHIKV/SFV chimeras

**Fig 1:**
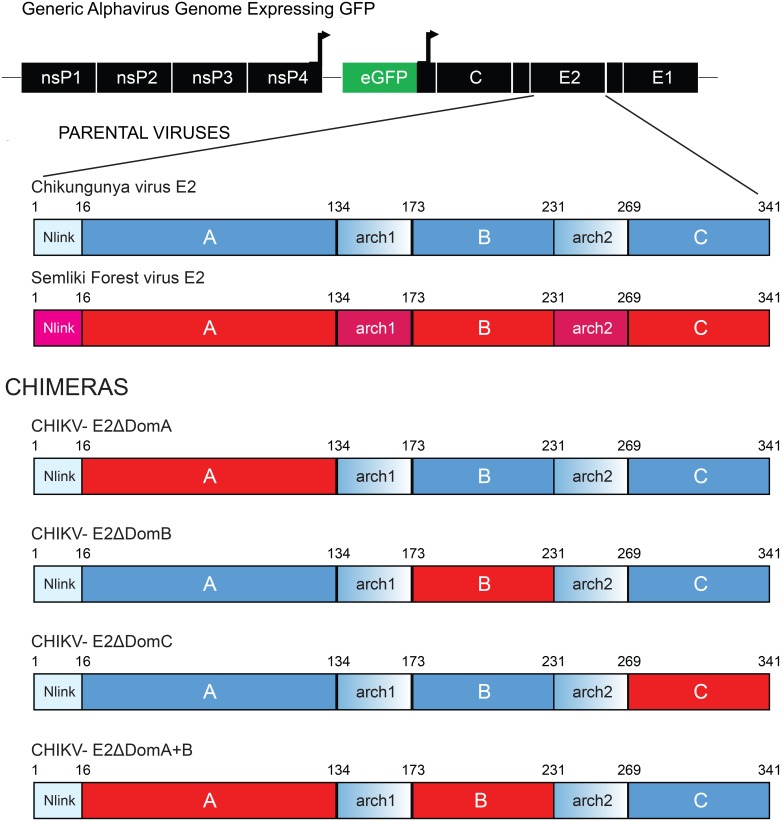
Genome organization of chimeric CHIKV/SFV viruses. Genome organization of chimeric CHIKV/SFV viruses. The different domains of E2 from SFV were inserted into the CHIKV genome in the corresponding position in individual constructs using a PCR based cloning approach. Each virus expressed the GFP protein under control of a second sub-genomic promoter. Red portions of the E2 represent genetic sequences of SFV whereas CHIKV is shown in blue.

CHIKV/SFV chimeras were constructed using a PCR-based cloning approach which allowed precise manipulation of DNA sequences without relying on restriction enzymes [27]. In total, four chimeric viruses were constructed (referred to as ΔDomA/ΔDomB/ΔDomC/ΔDomA+B) in which each domain(s) was replaced in a CHIKV backbone with the corresponding domain(s) from SFV (Fig 1). *In vitro* and *in vivo* characterization was performed and it was determined that each virus was viable in cell culture, mice and mosquitoes, although different phenotypes were observed (Weger-Lucarelli *et al*. manuscript submitted).

### Immunogenicity of live-virus in C57bl/6 mice

**Fig 2:**
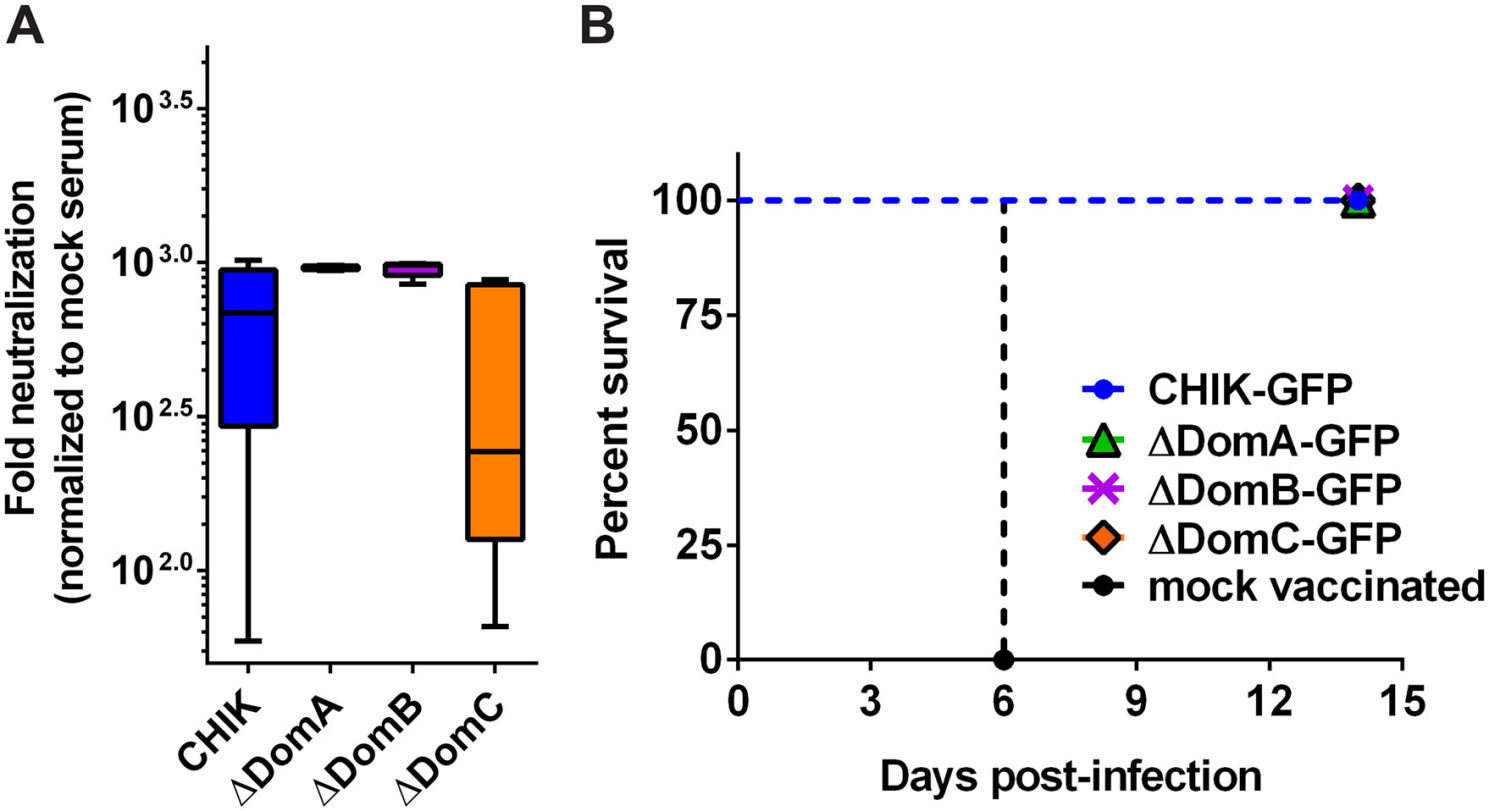
Protection and neutralizing antibody response elicited by chimeric CHIKV/SFV. Adult C57bl/6 mice (n = 6) were infected with 10^5^ PFU of CHIK, SFV, or chimeric viruses (ΔDomA, ΔDomB or ΔDomC) in the left hind footpad. Two months later, mice were bled for neutralizing antibodies and challenged with 10^5^ PFU SFV. A) Levels of neutralizing antibodies against SFV were measured by incubating serum from vaccinated mice with a SFV construct expressing nano-luciferase overnight at 4°C. The next day, the virus:serum mixture was used to infect BHK-21 cells in 96 well plates. After one hour adsorption period, cells were washed and fresh media was added. After five hours infection, cells were lysed and luciferase signal measured. Relative luminescence was normalized to a mock vaccinated control serum. SFV is not included as all infected mice rapidly succumbed to infection. B) Challenged mice were monitored for 15 days following infection. Data are expressed as percent survival.

C57bl/6 mice were selected for initial characterization of immune responses generated against CHIKV/SFV chimeras because they serve as an immunocompetent arthritis model for CHIKV [34] and a lethal encephalitis model for SFV [35]. Groups of mice (n = 6) were administered either viral diluent (mock infected) or 10^5^ PFU of each virus (except ΔDomA+B) in the hind left footpad (hereafter designated as vaccinated). Mice administered SFV succumbed rapidly, while all other groups remained healthy besides footpad swelling (Weger-Lucarelli *et al*. in revision). Serum samples from mice that were vaccinated with the chimeric viruses were assessed for neutralization against SFV using a luciferase-based assay [31], and there were no significant differences observed between mean neutralization titers against SFV when comparing ΔDomA or ΔDomB to CHIKV infected mice (One-way ANOVA with Tukey’s correction) (p = .13 and .15, for ΔDomA and ΔDomB respectively)) (Fig 2a). Serum from mice infected with ΔDomC had significantly reduced mean neutralization capacity as compared to either ΔDomA or ΔDomB (p<0.01 for both groups). However, serum from mice that were infected with chimeric viruses ΔDomA and ΔDomB did have a highly significant difference in variances (F test p<0.001) in neutralizing titers against SFV, indicating that both domains A and B are important for developing consistent neutralizing antibody responses.

**Fig 3:**
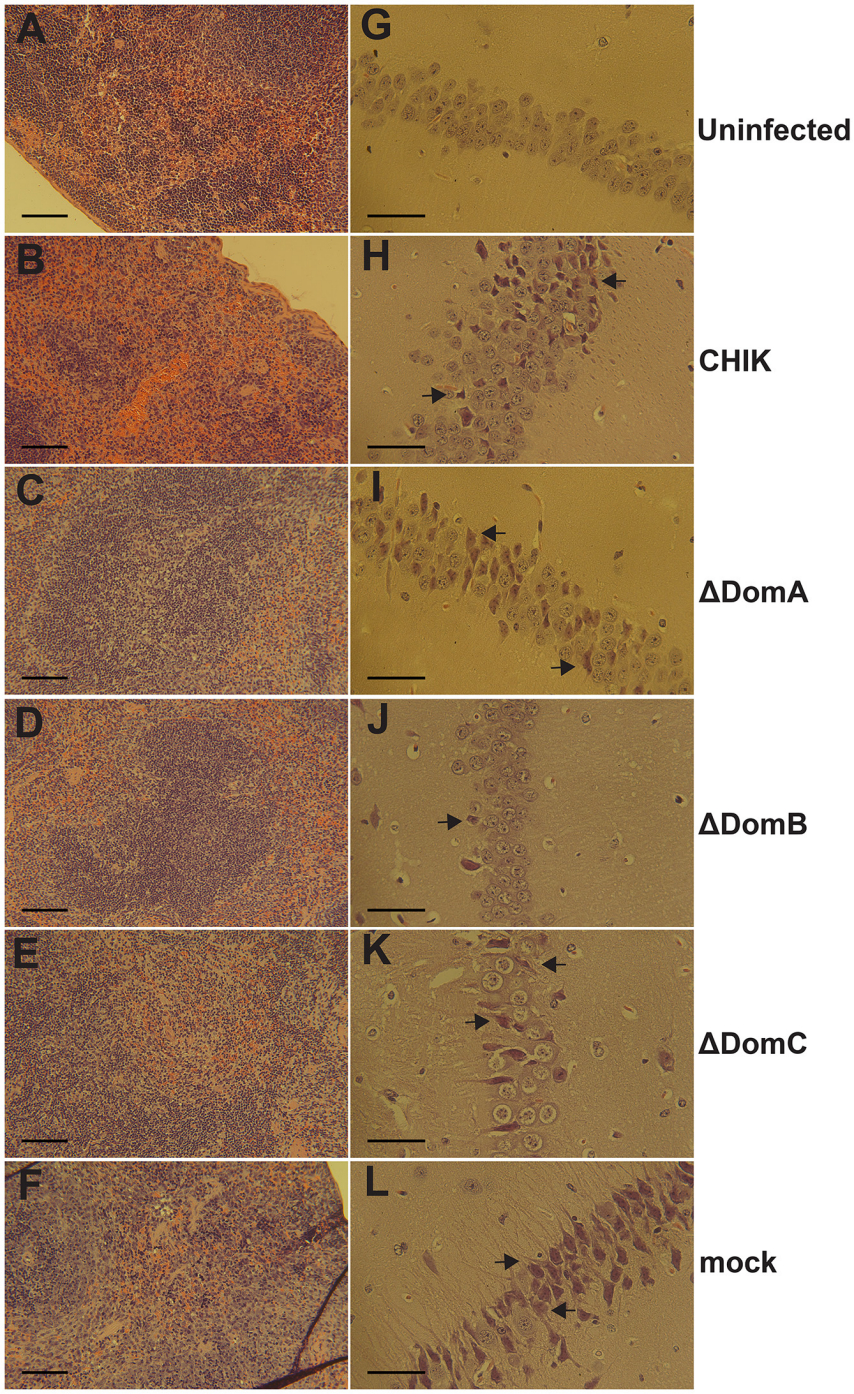
Representative histopathology of spleen and brain of live-virus vaccinated C57bl/6 mice post-challenge. Mice previously infected with 10^5^ PFU of CHIK or chimeric viruses (ΔDomA, ΔDomB or ΔDomC) were challenged with 10^5^ PFU of SFV. Surviving mice were euthanized and spleens and brains were harvested followed by processing for Hematoxylin and Eosin (H&E) staining. A-F. Spleens at 10x magnification. The scale bars represent 100 μM. G-L. Hippocampal neurons at 25x magnification. The scale bar represents 50 μM. Arrows signify neuron degeneration.

To assess the role of E2 domains in protection, vaccinated mice were challenged with 10^5^ PFU wild-type SFV via the same route two months post vaccination. To evaluate the protective efficacy of the chimeric viruses, mouse mortality was monitored following challenge. All mock vaccinated mice quickly succumbed to infection (Fig 2b). In contrast, all other mice survived challenge with no overt clinical signs. Because mice survived infection without overt clinical signs, we undertook a comparative histological analysis of the spleen and brain five days post infection, specifically surveying for obvious morphological changes as the result of secondary challenge. Based on previous literature, particular attention was paid to lymphocyte depletion in the spleen and degeneration of hippocampal neurons in the brain [36]. Examination of H&E sections of mouse spleen did not reveal obvious changes in splenic architecture or lymphocyte levels (Fig 3a) associated with inoculation of diluent alone. As compared to mock inoculated controls, pathology was detected in spleens of all mice vaccinated with chimeric viruses and then challenged with SFV, albeit to a lesser degree than mice that were mock vaccinated and then challenged with SFV (Fig 3b–3f). Spleens of mock-vaccinated mice exhibited massive lymphocyte depletion (Fig 3f). Mice vaccinated with CHIKV displayed moderate levels of lymphocyte depletion following SFV challenge (Fig 3b). In contrast, ΔDomA and ΔDomB-vaccinated mice experienced mild lymphocyte depletion after SFV challenge (Fig 3c and 3d). Examination of hippocampal neurons of challenged mice revealed that vaccination with ΔDomB appeared to protect mice from neuro-invasion of SFV, i.e., neurons were almost completely intact (Fig 3g–3j). Considerable lesions in the hippocampus were observed in all other groups and mock-vaccinated mice exhibited severe neuron degeneration in the hippocampus (Fig 3f).

### Immunogenicity of UV-inactivated viruses in C57bl/6 mice

**Fig 4:**
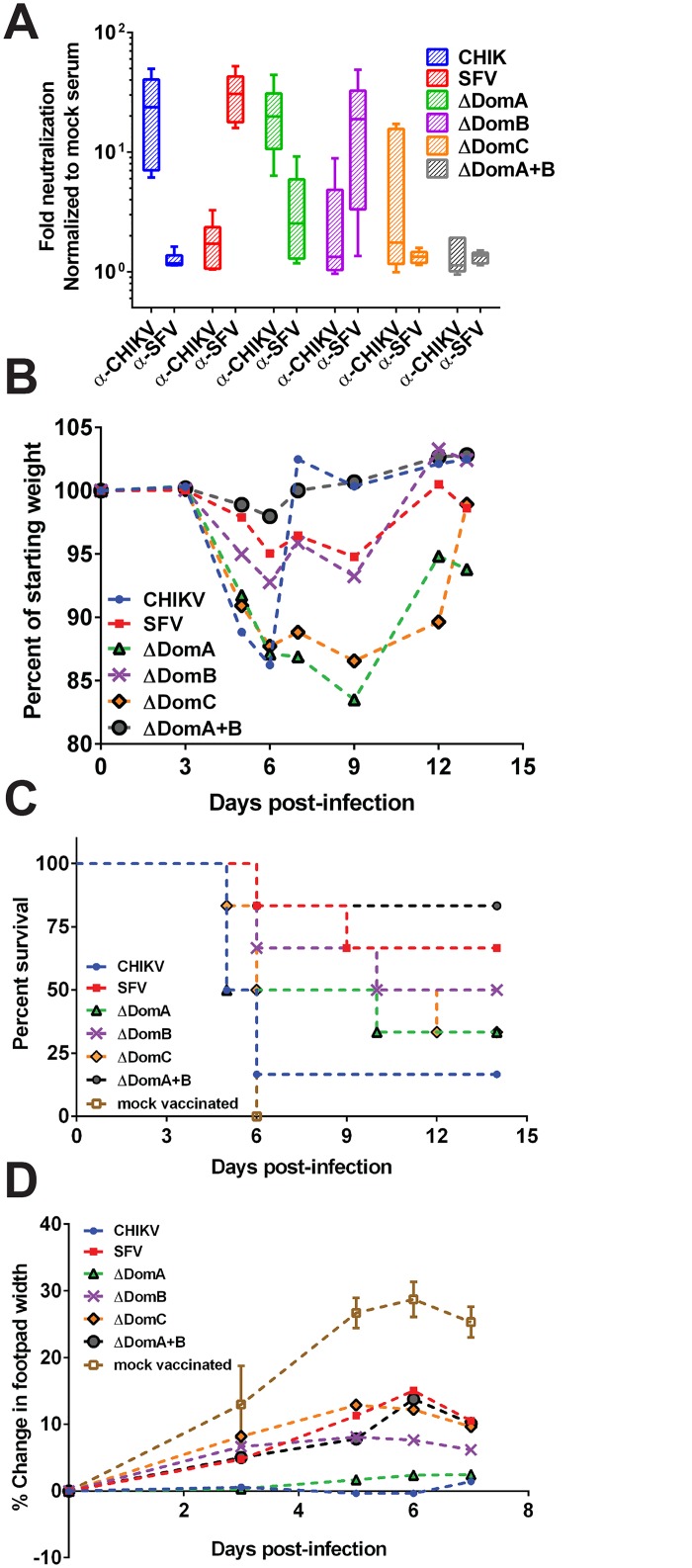
Neutralization and protection following vaccination with UV-inactivated viruses. Six-week old C57bl/6 mice were vaccinated with 5μg of each parental (CHIK or SFV) or chimeric viruses (ΔDomA, ΔDomB, ΔDomC, ΔDomA+B) inactivated with ultraviolet (UV) radiation. Mice received a second injection 28 days later. Groups of mice were then challenged with 10^5^ PFU of either CHIKV or SFV. A) Neutralizing antibody responses against both CHIK and SFV were assessed prior to challenge using a luciferase based assay. Infectious virus was mixed with a 1:20 dilution of serum and used to infect cells following incubation overnight at 4°C. Luciferase activity was measured in cell lysates after five hours of infection. Data are expressed as fold neutralization, normalized to mock serum. Mice challenged with SFV were monitored for B) weight loss and C) survival. Weight loss is expressed as the mean percentage of starting weight of the group. D) Change in footpad width was used as a marker of CHIKV disease. Data are presented as percent change as compared to pre-challenge levels.

In order to reduce the likelihood of other viral proteins and cell-mediated immunity confounding protection, we next vaccinated C57bl/6 mice with UV-inactivated virus. Groups of mice (n = 6) were immunized with 5 μg of inactivated virus in the left hind footpad followed by a second immunization 28 days later (boost). To determine the cross neutralization potential of mice vaccinated with inactivated viruses, mice were bled four weeks post-boost to measure levels of neutralizing antibodies against both CHIKV and SFV. Mice vaccinated with inactivated CHIKV or SFV displayed neutralization against homologous virus but very little cross-neutralization was observed (Fig 4a). Mice vaccinated with ΔDomA chimeric virus neutralized CHIKV but poorly neutralized SFV (p<0.01). In contrast, the ΔDomB virus showed a reverse pattern, losing much of its neutralization capacity to CHIKV while gaining significant neutralization to SFV (p<0.05). Neutralization was significantly reduced in mice vaccinated with either ΔDomC or ΔDomA+B viruses against CHIKV (p<0.05 and p<0.01) and neither virus produced detectable neutralizing antibodies to SFV.

Four weeks post-boost, mice were challenged with 10^5^ PFU of either CHIKV or SFV and monitored for two-weeks for signs of morbidity and mortality. Mice vaccinated with UV-inactivated CHIKV, ΔDomA or ΔDomC viruses quickly experienced weight loss after SFV challenge (Fig 4b). Although mice vaccinated with UV-inactivated SFV, ΔDomB, or ΔDomA+B experienced weight loss as well, peak reduction was not as drastic. As expected, all mock vaccinated control mice uniformly succumbed to SFV challenge (Fig 4c). Interestingly, despite producing low neutralizing antibody titers against SFV, 83% of the mice vaccinated with ΔDomA+B virus were protected from SFV challenge (p<0.05 as compared to UV-inactivated CHIKV vaccinated mice). Mice vaccinated with CHIKV were the least protected, with approximately 83% of the mice succumbing to SFV infection. There were no significant differences in survival observed in the other groups, as compared to mice vaccinated with UV-inactivated CHIKV. Mice challenged with CHIKV were monitored for footpad swelling as a marker of CHIKV-induced inflammation (Fig 4d). Mice vaccinated with inactivated CHIKV or ΔDomA were protected from footpad swelling, and experienced a significantly reduced change in footpad width compared to all other vaccinated groups for the duration of the study (p<0.05 at each time point tested). Mock vaccinated control mice displayed significantly greater change in footpad size than any of the vaccinated groups, suggesting that some protection against CHIKV-induced footpad swelling was elicited by all inactivated viruses (p<0.05 on days 5–7, compared to all other groups).

**Fig 5:**
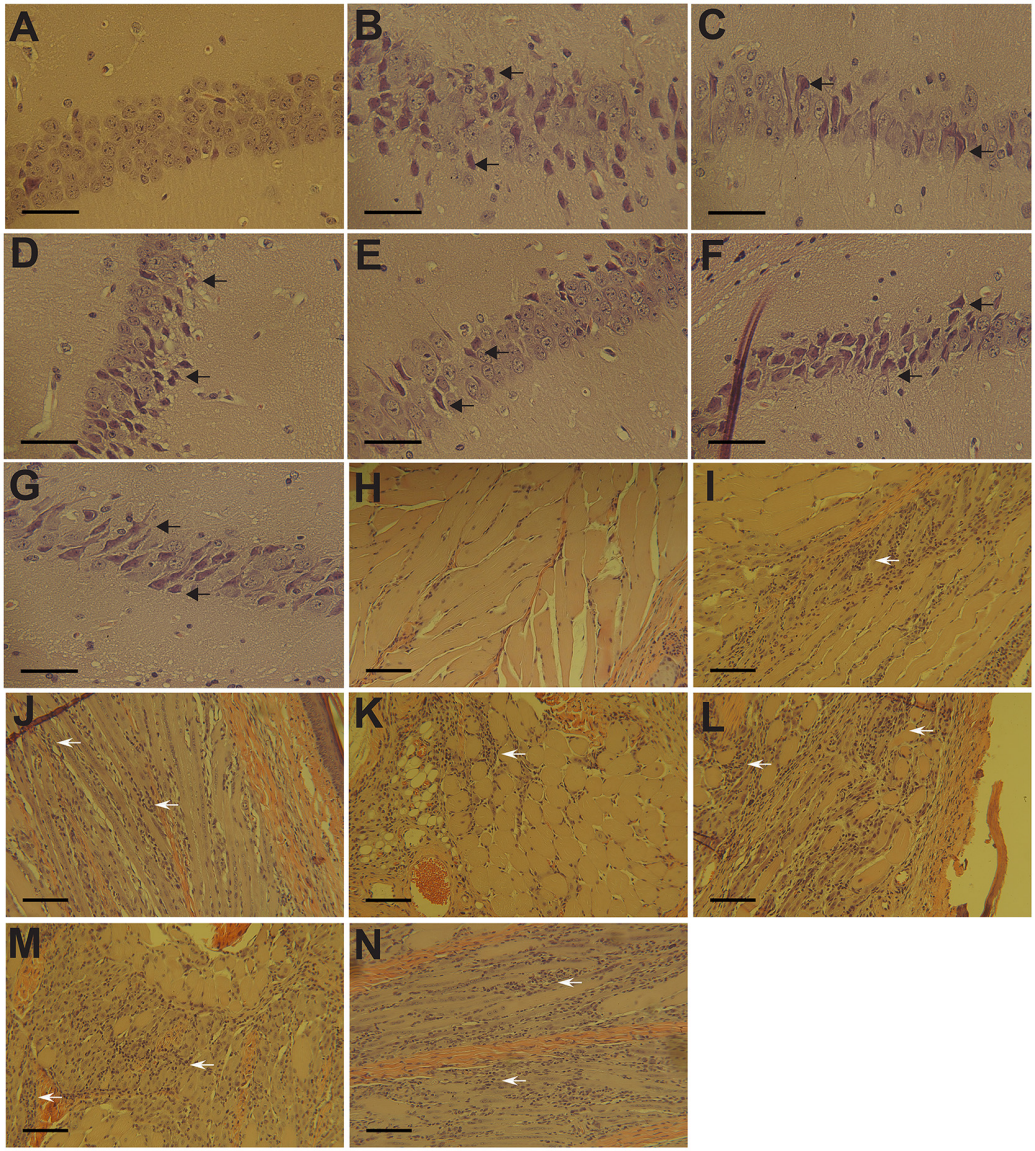
Protection afforded by UV-inactivated parental or chimeric viruses. Groups of mice previously vaccinated with 5μg prime and boost of each UV-inactivated parental (CHIKV or SFV) or chimeric (ΔDomA, ΔDomB or ΔDomC, ΔDomA+B) virus were challenged with 10^5^ PFU of either wild-type CHIKV or SFV. Five days post-infection, 3 mice from each group were euthanized and brains and footpads were harvested for SFV and CHIKV challenge groups, respectively. Tissues were processed for hematoxylin and eosin (H&E) staining. A-G. Hippocampal neurons from SFV challenged mice using a 25x objective. (A) Uninfected control, (B) CHIKV, (C) SFV, (D) ΔDomA, (E) ΔDomB, (F) ΔDomC, (G) ΔDomA+B. Scale bars represent 50 μM. Black arrows signify neuron degeneration. H-N. Decalcified footpads of CHIKV challenged mice using a 10x objective. (H) Uninfected control, (I) CHIKV, (J) SFV, (K) ΔDomA, (L) ΔDomB, (M) ΔDomC, (N) ΔDomA+B. Scale bars represent 100 μM. White arrows represent areas of mononuclear cell infiltration into the tissue.

Five days after challenge, three mice from each group were sacrificed to monitor histopathological changes in the brain and footpad for SFV and CHIKV-challenged groups, respectively. Mice administered diluent only maintained intact hippocampal neurons, as expected (Fig 5a). In contrast, mice vaccinated with inactivated CHIKV displayed moderate to severe neuron degeneration in the hippocampus following SFV challenge (Fig 5b). SFV-vaccinated mice consistently showed little neuron degeneration in the same region (Fig 5c). In addition, groups vaccinated with ΔDomA, ΔDomC or ΔDomA+B viruses exhibited moderate-to-severe neuron degeneration, similar to CHIKV-vaccinated mice (Fig 5d–5g). Mice immunized with ΔDomB, however, demonstrated mild hippocampal neuron degeneration following SFV challenge, consistent with SFV-vaccinated mice (Fig 5e).

Vaccinated mice challenged with CHIKV displayed a highly different infection outcome as compared to those mice challenged with SFV. Mice receiving diluent alone did not develop significant inflammation in the footpad, as anticipated (Fig 5h). Mild mononuclear cell infiltration and myositis were observed in footpads of CHIKV or ΔDomA vaccinated mice (Fig 5i and 5k). In contrast, mice in groups vaccinated with SFV, ΔDomB, ΔDomC, ΔDomA+B had moderate to severe myositis with increased inflammatory cell infiltration in the tissue (Fig 5j–5n). These data were consistent with neutralizing antibody titers elicited by vaccination, as all of these groups had significantly reduced neutralization capacity to CHIKV, as compared to UV-inactivated CHIKV vaccination (Fig 4a).

### Neutralization of parental and chimeric viruses by human sera

**Fig 6:**
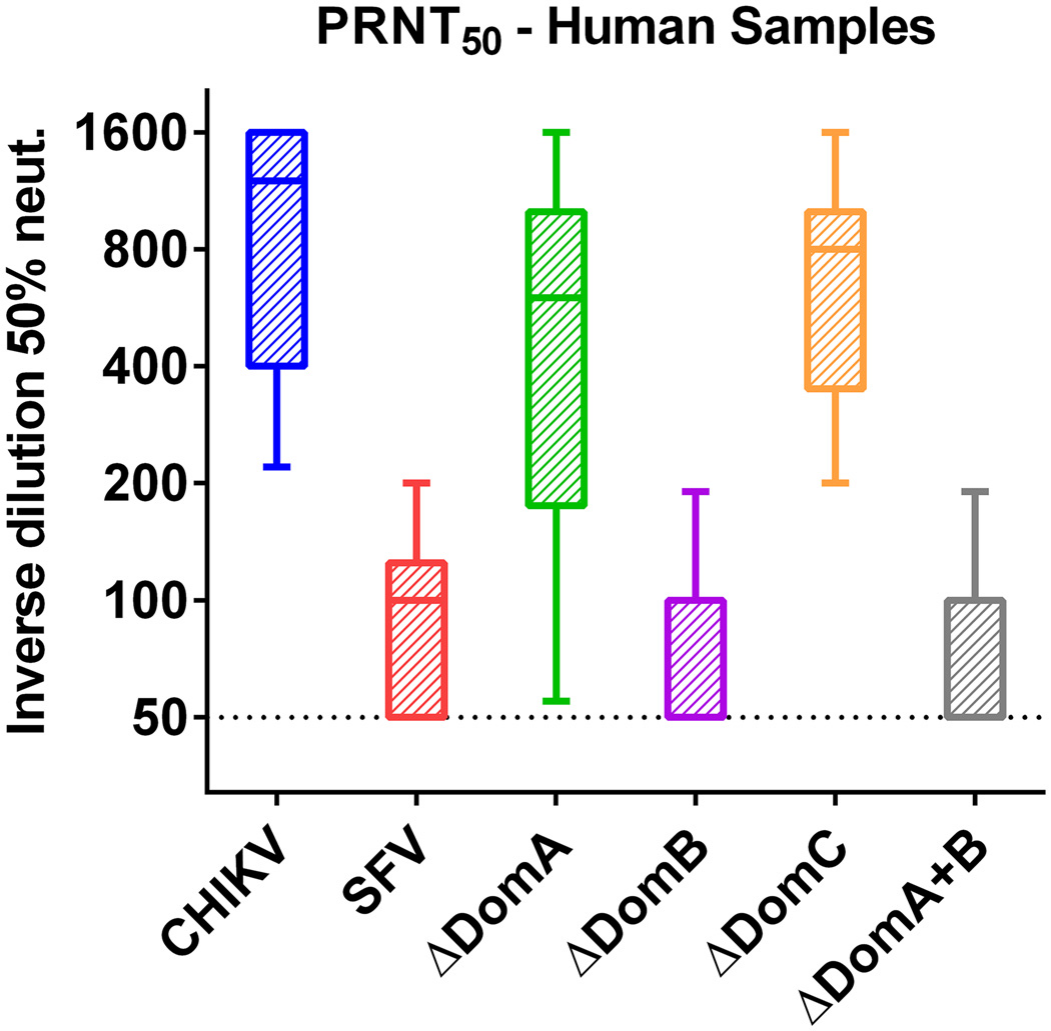
Neutralization of parental and chimeric viruses with CHIKV-immune human serum. Convalescent serum samples from 10 human patients were collected aseptically. Neutralization was performed using plaque reduction neutralization 50 (PRNT50) test. Two-fold dilutions of serum samples were incubated individually with each parental (CHIKV or SFV) or chimeric virus (ΔDomA, ΔDomB or ΔDomC, ΔDomA+B). Cells were fixed 36 hours post-infection and stained with crystal violet solution to visualize plaques. Data is presented as the inverse of the final serum dilution that showed 50% reduction or greater in plaques.

In order to validate our work in mice, convalescent human serum samples obtained from patients previously infected with CHIKV were tested for neutralization capacity of each of the parental (CHIKV and SFV) or chimeric viruses (ΔDomA/ΔDomB/ΔDomC/ΔDomA+B). Samples were isolated from 10 volunteers during the current outbreak from Martinique or Colombia. As expected, the highest neutralization titers were observed against CHIKV, while little to no neutralization was observed against SFV (Fig 6). ΔDomA and ΔDomC viruses had high neutralizing titers against CHIKV. In contrast, ΔDomB and ΔDomA+B were neutralized at a significantly lower capacity than CHIKV or ΔDomA (p<0.01). These data indicated that domain B is critical for effective neutralization of CHIKV.

## Discussion

The massive ongoing outbreak and global spread of CHIKV has highlighted the need for a vaccine against this virus. The development of a vaccine is hampered by the lack of knowledge of specific domains of protection that can assist in designing rationale vaccines that are safe and highly effective. Recombinant live-attenuated (LAV) or sub-unit vaccines that target a particular domain of CHIKV might represent the best option for a vaccine candidate. We have previously shown that an attenuated poxvirus, modified vaccinia Ankara (MVA), expressing only CHIKV E3 and E2 proteins was a safe and effective vaccine candidate [21]. Other groups have shown that E2 or peptides within E2 can produce a protective immune response in mice [13, 37, 38]. A recent study showed that neutralizing antibodies and protection could be induced by vaccination with sub-unit antigens consisting of either domain B or domains A and B together, suggesting individual domains of the receptor binding protein are sufficient to produce a neutralizing antibody response [39].

Here, we attempted to characterize the role of each of the E2 domains of CHIKV for protection and immunogenicity in mice. We constructed a panel of chimeric viruses between CHIKV and the closely related Semliki Forest virus (SFV). Each virus contained a different domain of CHIKV E2 (referred to as, ΔDomA, ΔDomB, or ΔDomC) replaced with the corresponding domain of SFV. An additional virus was constructed that contained both domains A and B from SFV (called ΔDomA+B). SFV was selected because it is sufficiently similar to CHIKV to produce viable chimeric viruses, while previous literature suggests that it is not neutralized by CHIKV immune serum [26]. While data generated in this report suggest that anti-CHIKV serum can neutralize SFV as well, this may have been due to differences in assay, virus strains, mouse strains, or a variety of other factors, as the previous report was published in 1961. To our knowledge, this is the first study to examine the role of alphavirus E2 domains in the context of a live virus that maintains interactions with E1 and capsid, which have been shown to be important for proper protein folding and viral assembly [40, 41]. Previous studies have shown that many of the determinants of neutralizing antibodies, host range and tissue tropism reside with domains A and B of the E2 (reviewed in [10]); therefore, we hypothesized that these domains would be the primary determinants of protection.

To test our hypothesis, mice were first infected with live parental CHIKV or chimeric viruses. All groups of mice produced neutralizing antibodies and survived highly lethal challenge with SFV. This was expected as previous reports have shown cross-protection between highly divergent alphaviruses [42–44]. Mice vaccinated with ΔDomA or ΔDomB produced significantly reduced variability in neutralizing titers than mice vaccinated with CHIKV or ΔDomC. This suggested that transferring domains A or B from SFV in to a CHIKV backbone could augment the cross-protective immune response observed. However, when organs of challenged mice were examined for SFV-induced pathology it was determined that ΔDomB vaccination reduced both neuro-invasion and lymphocyte depletion (as compared to CHIKV-vaccinated mice) caused by SFV. While lymphocyte depletion appeared to be reduced in ΔDomA-vaccinated mice (as compared to CHIKV-vaccinated mice), it did not prevent moderate to severe neuron degeneration in the hippocampus that was also seen in CHIKV vaccinated mice. This suggested that the immune response against domain B is important to restrict SFV spread and replication. In addition, vaccination with UV-inactivated ΔDomB virus resulted in high neutralizing antibodies against SFV but not CHIKV, while ΔDomA virus produced the opposite. Furthermore, mice vaccinated with UV-inactivated ΔDomB exhibited reduced pathology upon challenge with SFV but were not protected against CHIKV challenge. Conversely, mice vaccinated with UV-inactivated ΔDomA were protected against severe CHIKV pathology but not against SFV challenge. While mice vaccinated with inactivated ΔDomC virus produced neutralizing antibodies against CHIKV, but not SFV, they were not protected against CHIKV induced myositis following challenge. Also, mice vaccinated with inactivated ΔDomA+B did not produce neutralizing antibodies against either virus, nor was protection observed. This was likely due to improper folding of the E1/E2 glycoprotein complex on the surface of the virion through disruption of important viral envelope protein interactions. It has previously been shown that the interaction between E1 and E2 is important for proper assembly of the alphavirus glycoprotein complex [10, 45]. Therefore, it is probable that substitution of wild-type CHIKV sequences with those from SFV would disrupt protein folding and assembly in a manner which could alter the host immune response. Interestingly, development of neutralizing antibodies against SFV did not correlate to protection from lethal SFV challenge as similar survival rates were observed between the groups. Previous reports have shown that cell-mediated immunity is important for protection against lethal challenge with SFV and that protection could be conferred by adoptive transfer of immune spleen cells [46, 47]. Vaccination with UV inactivated virus in the absence of adjuvant would not be expected to induce a strong cell-mediated immune response and is possibly the cause of reduced survival. In addition, the dose used for SFV challenge was high, in the order of 1000 to 10000 LD_50_ according to other published reports [48]. The dose used in the study likely overwhelmed the immune response developed by vaccination with inactivated virus without adjuvant. It is probable that the addition of an adjuvant or a higher dose of protein would provide 100% protection. Finally, human serum from patients previously infected with CHIKV was able to neutralize CHIKV, ΔDomA and ΔDomC, but had lost the ability to neutralize either ΔDomB or SFV. Taken together, we conclude that E2 domain B is the primary mediator in the development of neutralizing antibodies. Mutations that are likely involved in cellular receptor binding are located on the exposed surfaces of the virion, mostly in domains B and A [10]. In addition, domain B functions to cap the fusion loop in the E1 protein [10]. The dual roles of domain B likely contribute to its critical role in the host immune response and development of neutralizing antibodies.

It is unclear why vaccination with live virus, but not UV-inactivated virus, resulted in equal levels of neutralizing antibodies for mice vaccinated with ΔDomA and ΔDomB. Likely, in the absence of an adjuvant, vaccination with inactivated virus was less robust than that with live virus. Replication of live virus is likely to induce the production of pro-inflammatory cytokines and result in further exposure of the immune system to viral antigen; resulting in increased levels of neutralizing antibodies. This is supported by the overall higher levels of neutralizing antibodies in mice vaccinated with live-virus as compared to inactivated. It has previously been shown that vaccination with live and inactivated virus vaccines result in different immune responses. For example, live-attenuated influenza viruses stimulate different antibody subtypes and pro-inflammatory cytokines than inactivated versions, resulting in increased heterosubtypic immunity [49–51]. Additionally, immunization with both inactivated and live-attenuated bovine respiratory syncytial virus vaccines induced similar levels of antibody to the F protein; however, only the latter vaccination strategy resulted in neutralizing antibodies [52]. This indicates that vaccination with inactivated antigen can result in highly different antibody specificity, consistent with the data presented in Figs 2 and 4. Taken together, it is not surprising that vaccination with live virus resulted in a stronger and more protective immune response than with unadjuvanted, inactivated vaccination.

Our studies highlight the importance of the alphavirus E2 domain B in the host immune response, likely acting as the primary target for neutralizing antibodies. Therefore, future vaccine candidates against CHIKV should focus on producing a strong antibody response to domain B. Interestingly, the ΔDomA and ΔDomC viruses reported in this paper are highly attenuated in both immunocompromised and immunocompetent mice; losing lethality in the former and showing significantly reduced replication and pathology in the latter (Weger-Lucarelli *et al*. in revision). These viruses thus represent candidates as rationally designed live-attenuated vaccine candidates. Both viruses maintain CHIKV E2 domain B and provide full protection against SFV (Fig 2) when given as live virus. This approach might represent a safer alternative to traditional live-attenuated vaccines, which rely on only one or two attenuating mutations for safety. Furthermore, immune responses against domain B may represent a useful marker for protective immune responses in vaccine or epidemiology studies.
